# What Have We Learned from 40 Years of Supporting Research and Capacity Building?

**DOI:** 10.1371/journal.pntd.0003355

**Published:** 2015-01-08

**Authors:** John C. Reeder, Jamie A. Guth

**Affiliations:** Special Programme on Research and Training in Tropical Diseases (TDR), a co-sponsored programme of UNICEF/UNDP/World Bank/WHO, based at the World Health Organization, Geneva, Switzerland; Lindsley F. Kimball Research Institute, New York Blood Center, United States of America

From the beginning of the 20th century, the industrialized “North” made incredible improvements in public health that reduced the threat of infectious diseases and increased overall health. Unfortunately, this was not the case in many other countries [1]. By the 1970s, major disease-control initiatives in low- and middle-income countries, which were driven vertically by donors, were having mixed results, and private-sector pharmaceutical firms had little incentive to invest in the drugs and tools needed by countries that could ill afford the research and development (R&D) costs.

In April of 1974, the 27th session of the World Health Assembly called for the “intensification of activities in tropical disease research” and the “strengthening of research and training activities”, particularly in developing countries [2]. Within two months, the World Health Organization (WHO)Advisory Committee on Medical Research met and discussed the need to “coordinate and stimulate biomedical research through bilateral and multinational arrangements,” and to apply “advances in knowledge in basic biology to urgent medical and public health problems.” They recommended an “expanded WHO programme for research and training related to tropical communicable diseases” [2].

By November of that year, TDR, the Special Programme for Research and Training in Tropical Diseases, was in operation. The main principles underpinning the formation of TDR are just as relevant today as they were when the Programme was established: to promote and conduct research equitably and to provide access to this knowledge and the resulting tools to the most vulnerable and hard-to-reach people.

It has been 40 years since that beginning, and many lessons have since been learned. This special collection of seven articles (including this one) is designed to share those lessons—what worked, what did not, and how the Programme has evolved to meet the changing needs of both researchers and the research fields. What has changed is the type of research supported, the way it was conducted, and even the diseases covered. As the needs in the countries evolved, so too did the Programme, which is explained in more detail in the following articles.

## Institutional Organization

TDR was initially sponsored by WHO, but the United Nations Development Programme (UNDP) was involved from the beginning and joined formally as a co-sponsor in 1976, followed by the World Bank in 1977 and the United Nations Children's Fund (UNICEF) in 2003. All organizations were committed to some aspect of infectious diseases, and it was agreed that working together in a co-sponsorship model would be more efficient. This has given the organization a broad UN platform from which to work.

Another innovation was the equal representation of donor and recipient governments on TDR's governing body, the Joint Coordinating Board (JCB), which was created in 1978 [3]. This gavepolitical credibility to any decisions made, and a mandate and commitment from disease-endemic countries.

Meanwhile, the Scientific and Technical Advisory Committee (STAC), an independent technical-oversight body, enlisted leading scientists, not only from global centres of expertise but also from disease-endemic regions and industry. This group has provided a strong scientific foundation for the work and anchored it firmly in the needs of the endemic countries' scientists [4].

We argue that this governance model and placement within the United Nations system has had great positive impact historically, and offers continuing value in the coming years. There are those who would argue that a UN agency is too bureaucratic and politically constricted, but it has been an ideal placement to set up a continuous loop of learning from control to research to control. The connection to WHO, UNDP, and UNICEF country and regional offices provides strong networks into the countries and a neutral platform where multiple partners can come together.

## Evolving Strategies for Research and Capacity Building

TDR's initial focus was on eight of the most neglected tropical diseases—malaria, leprosy, schistosomiasis, leishmaniasis, onchocerciasis, lymphatic filariasis, Chagas disease, and human African trypanosomiasis (HAT).

The needs were great, ranging from basic understanding of the parasites, vectors, and drug resistance, to developing new drugs, diagnostics, and strategies that could prevent and treat a range of infectious diseases. Underpinning this was the huge gap in skills and infrastructure in the countries where the diseases were endemic.

From this big wish list, TDR started with a focus on basic science. Malaria would eventually draw the largest resources [5], with funds first directed at investigating the parasite, looking at vector biology and how immunity works. There was also work on genomics and the genetic modification of the vectors for malaria and dengue [6] and establishing chemical compound screening networks to identify leads for new drugs and diagnostic tools [7].

Within the first few years, it was clear that this type of new knowledge, while valuable, would take decades to complete and still not directly improve health conditions in these endemic countries. So TDR also moved into more product-based research that offered additional opportunities for scientists from the low- and middle-income countries to get involved, since these products had to be tested in their own countries [7]. This approach helped bring online new treatments for human African trypanosomiasis, leprosy, malaria, and onchocerciasis.

At the same time, barriers to accessing these new products were identified, which helped drive complementary social and health systems research. This led to colored bednets (which were more acceptable to the community than the original white ones), better designed packaging for malaria treatments, and community models for both malaria care and mass treatment for onchocerciasis—changing from a donor and northern-driven approach to a bottom-up, community empowerment approach [8].

One key lesson learned has been that it takes time to develop sustainable research capacity, and it requires local and national involvement and commitment. Up until 2000, about a third of TDR's total resources were earmarked to strengthen research capacity in low- and middle-income countries. The goal was to eventually have priorities set and research conducted by people living in the countries where the diseases were endemic. So TDR developed programmes to build institutions and also support individuals through degree programmes and individual courses [9].

More than 1,400 postgraduate training grants were awarded between 1975–1996 (a third of these to those from the lowest-income countries). Many of these grantees are now leading and building research organizations in their countries, as well as providing lead technical support in ministries of health and international organizations, including WHO. A significant number specialized in malaria and went on to make major contributions to the field.

Networks were created to connect researchers in the “South” with those in the high-income countries, and also with those in other low- and middle-income countries. Of particular note was the South–South Initiative that drew together researchers across Africa, Latin America, and Asia. The goal was to build capacity through networking, shared training, and the development of common protocols for basic research in pathogenesis and genomics [10].

The question now facing TDR is how to support today's researchers and strengthened institutions. How can this momentum continue, and are there different ways of learning and support that should be provided? People can now get their PhDs and specialized training at institutions in their own countries, rather than having to go to United States or European institutions. What they ask from TDR today is help in making the links between evidence and policy and change, so support is more flexible and tailored to their needs, rather than a one-size-fits-all approach to degree programmes and specific courses. The support has expanded from core scientific training (which continues for new scientists) to helping scientists who are already established build their career and their institutions. They need research for scaling up of new health products and strategies, and methods for prioritizing research needs and connections beyond their national borders. An alumni network is being developed that will better track people, assess the value of current support structures, and help connect people to information and groups [9].

We have learned that increasing the capacity of countries is not only about financial and political commitment but about ongoing mentorship and growing a network.

## Research Contribution to Disease Control and Elimination

Five of the eight diseases that were originally targeted by TDR are moving towards regional or global elimination (Chagas disease, leprosy, lymphatic filariasis, onchocerciasis, and visceral leishmaniasis). Each of these required strong partnerships with national governments, research institutions, and local researchers to generate the solid research results that have been game changers.

Due in part to the evidence TDR generated on the effectiveness of multidrug therapy, the incidence of leprosy went down from 5.2 million registered cases in 1985 to 220,000 in 2006 [11].

A 1998 World Health Assembly resolution confirming the interruption of Chagas disease transmission in several countries in Latin America followed large-scale elimination campaigns in the 1990s. TDR supported epidemiological surveys, vector-control tools, and blood screening that helped lay the foundation for these elimination campaigns.

TDR collaborations helped provide the evidence that a single dose of diethylcarbamazine (DEC) treatment was as effective as a longer treatment, helping pave the way for the creation of the Global Programme to Eliminate Lymphatic Filariasis in 2000. Additional studies documenting the economic cost played a critical role in stimulating the interest of national policy-makers to address the issue [12].

River blindness is now under control in most of Africa, in part thanks to a long-term partnership between TDR and the African Programme for Onchocerciasis Control (APOC), which identified the needs and worked with TDR to support and implement the results. This evolved into studies of community-based models of care that have provided evidence that community health workers can support numerous health conditions, like malaria diagnosis and treatment, and bednet and vitamin A distribution [13].

The collaborations to develop new drug treatments and management strategies have helped drive the elimination of visceral leishmaniasis programs in Bangladesh, India, and Nepal. TDR has brought high-level representatives of the countries together to identify research needs and develop both shared and individual strategies going forward. They have collectively developed novel approaches to controlling the sandfly vectors that transmit the disease, and an effective strategy for point-of-care diagnosis and treatment close to endemic villages [4].

TDR has provided key support to improving malaria treatments and access to them. Milestone research provided the evidence on the effectiveness of insecticide-treated bednets to reduce malaria transmission. Bednets are now one of the key elements of malaria control throughout Africa. The first artemisinin-combination treatment was tested by TDR and shown to be efficacious and cost effective, the safety of its use in very young children weighing as little as 5 kilograms was established [14], and unit-dose packages were shown to improve adherence to treatment and reduce costs [15]. Access to malaria treatments has increased in many African communities due to research demonstrating the effectiveness of community health workers (CHWs) to diagnose and treat malaria within the first 24 hours, a key time period for children, who can become seriously ill and die. This concept of using CHWs was further explored with research on community-based integrated management of childhood fevers, which includes malaria, pneumonia, and diarrhoea [16], which led to a joint WHO/UNICEF statement calling for scaling up this approach [17].

Evaluations of rapid diagnostic tests have helped countries to diagnose and monitor several critical diseases, including tuberculosis, malaria, dengue, and visceral leishmaniasis. Nine syphilis tests that were shown to be effective were placed on the WHO procurement list, allowing countries to purchase these at reduced prices and introduce them as standard tests among pregnant women, who could carry syphilis with no symptoms and pass it on to their unborn baby. As a result, several countries now have national elimination plans [14].

TDR's extensive vector research has identified numerous solutions to prevent disease transmission, including improved traps and targets to catch the tsetse fly of African trypanosomiasis; the use of indoor residual spraying (on interior house walls), insecticide-treated netting materials and environmental management to reduce the number of *Aedes* mosquito-breeding sites and dengue transmission; and impregnated window curtains and screens to reduce the numbers of triatomine bugs and Chagas disease.

The best results, we have learned, come from equitable partnerships with control programs and local government, where each learns from the other during long-term commitments. A key lesson in all this work is the value of working closely with WHO and national health officers so that control could inform what kind of research evidence was needed and policy officers could update guidelines based on evidence and work with the countries to help implement these changes.

## Moving Forward—Making an Impact

Global health research and development dramatically increased in the late 1990s, with much of this focused on new drugs, diagnostics, and vaccines [18]. TDR's budget remains modest, but the way it has been invested has provided important and long-lasting public health impacts, and we would argue, one of the best values in R&D to address diseases of poverty. The majority of TDR funding comes from bilateral overseas aid programmes, in recognition of the link between health and development (with research being a prerequisite for health). It's this core funding that helps us set the agenda, rather than react to funding trends.

The early years were investments to build the numbers of researchers and institutions and to increase knowledge from basic science that could be used later for new tools and strategies. We now have new types of training support where the grantees identify specific skills they can′t get in a traditional programme that will allow them to make faster changes, and we′re expanding regional training centres to imbed this support closer to home.

TDR also revised its research strategy, focusing more resources on research that could be implemented more quickly with a trained and available research body. The vector research strategy is a good example of this [6], in which funding moved from investigator-driven vector genomics to country-driven needs to prevent disease transmission.

TDR now focuses more deeply on implementation and operational research that increases access to the products and strategies already available and shown to work. We have found that creating good health products does not automatically guarantee their distribution and use. Sometimes there are issues of gender or cultural appropriateness, often there are system weaknesses, and consistently, we have shown the need for involvement at the community level.

The World Health Report 2013 [19] called for more low- and middle-income countries to be not only users but producers of health research. We could not agree more. To address system bottlenecks and get research solutions taken up more rapidly, we are leading a collaboration called SORT IT [20] that provides research and training support to public health programmes in low- and middle-income countries. We have also published an implementation research toolkit [21] for use by a broad range of stakeholders that is the product of consultations with more than 200 people and numerous pilot workshops.

In the following articles of this collection, former and current TDR staff provide their views on key challenges and lessons learned during the 40-year history, and they explain how and why the approaches and workplans changed through time. What is clear is that the TDR principles, which have remained stable throughout this evolution, provided important underpinnings to allow this flexibility, and eventually, the successes.

TDR principles:

- Research carried out in the countries where the diseases occur and by scientists from those countries.
- Long-term commitment to strengthening research capacity.
- Partnership model—for identifying research priorities, setting up multidisciplinary projects, and managing these projects.
- Organizational sponsorship and structure within the UN system, and a governance model that provides equitable input from low-, middle-, and high-income countries.
- Continual assessment of progress and flexibility in changing the strategy and priorities.
